# Viral vector-based transient expression systems for plant biotechnology research at PUIs

**DOI:** 10.3389/feduc.2025.1598673

**Published:** 2025-06-01

**Authors:** Kevin Wang, Kylie Hall, Kylie Tackett, Holly Jordan, Gabriella Hall, Peyton Campbell

**Affiliations:** Division of Math and Natural Sciences, College of Arts & Sciences, University of Pikeville, Pikeville, KY, United States

**Keywords:** undergraduate research, plant transient expression, research curriculum, semester/term-based research, low cost, accessibility for PUIs, rapid experimental turnaround, educational value

## Abstract

Traditional stable genetic transformation in plant biotechnology remains largely inaccessible at many Primarily Undergraduate Institution (PUIs) due to high costs, long timelines, and specialized facility demands. Viral vector-based transient expression systems offer an efficient and accessible alternative method that enables meaningful undergraduate research within a single academic term. These systems utilize plant virus-derived vectors (e.g., TMV or Geminivirus) to transiently express target genes, producing detectable recombinant proteins within 3–7 days. Requiring only basic lab tools, they align well with Course-based Undergraduate Research Experiences (CUREs), lab courses, and capstone projects. Students gain practical experience in gene cloning, agroinfiltration, protein or metabolite chemical analysis, while faculty benefit from increased research capacity and funding potential. This mini-review highlights the advantages, implementation strategies, and funding opportunities of viral vector-based transient expression systems at PUIs, underscoring their value in expanding access to synthetic biology, plant-based biomanufacturing, and interdisciplinary STEM education.

## 1 Introduction

Plant biotechnology is a rapidly advancing that integrates genetic engineering, molecular sciences and applied chemistry to address major global challenges in agriculture, environmental sustainability, and human health (Fernie and Sonnewald, 2021; Halpin et al., 2023). Advances in recombinant protein production, metabolic engineering, and synthetic biology have enabled plant systems to serve as platforms for the generation of therapeutic proteins, vaccines, industrial enzymes, and biofuels. As priorities such as food security, climate resilience, and public health drive research, there is a growing demand for a biotechnology workforce equipped with hands-on training in plant molecular technologies (Buyel, 2024).

Primarily Undergraduate Institutions (PUIs) play a pivotal role in cultivating the next generation of scientists and biotechnologists by offering students early exposure to authentic research. However, they often encounter barriers to implementing plant biotechnology programs due to limited funding, restricted laboratory infrastructure, and the requirement to align experimental timelines with academic semesters (Wang, 2017).

A key constraint in traditional plant biotechnology is its reliance on stable genetic transformation, a process that can take several months to years to generate transgenic plants from sterile tissue culture (Su et al., 2023). This technique demands substantial resources, including tissue culture facilities, controlled growth chambers, and specialized technical skills (Table 1). This complexity makes it impractical for most PUIs.

**FIGURE 1:**
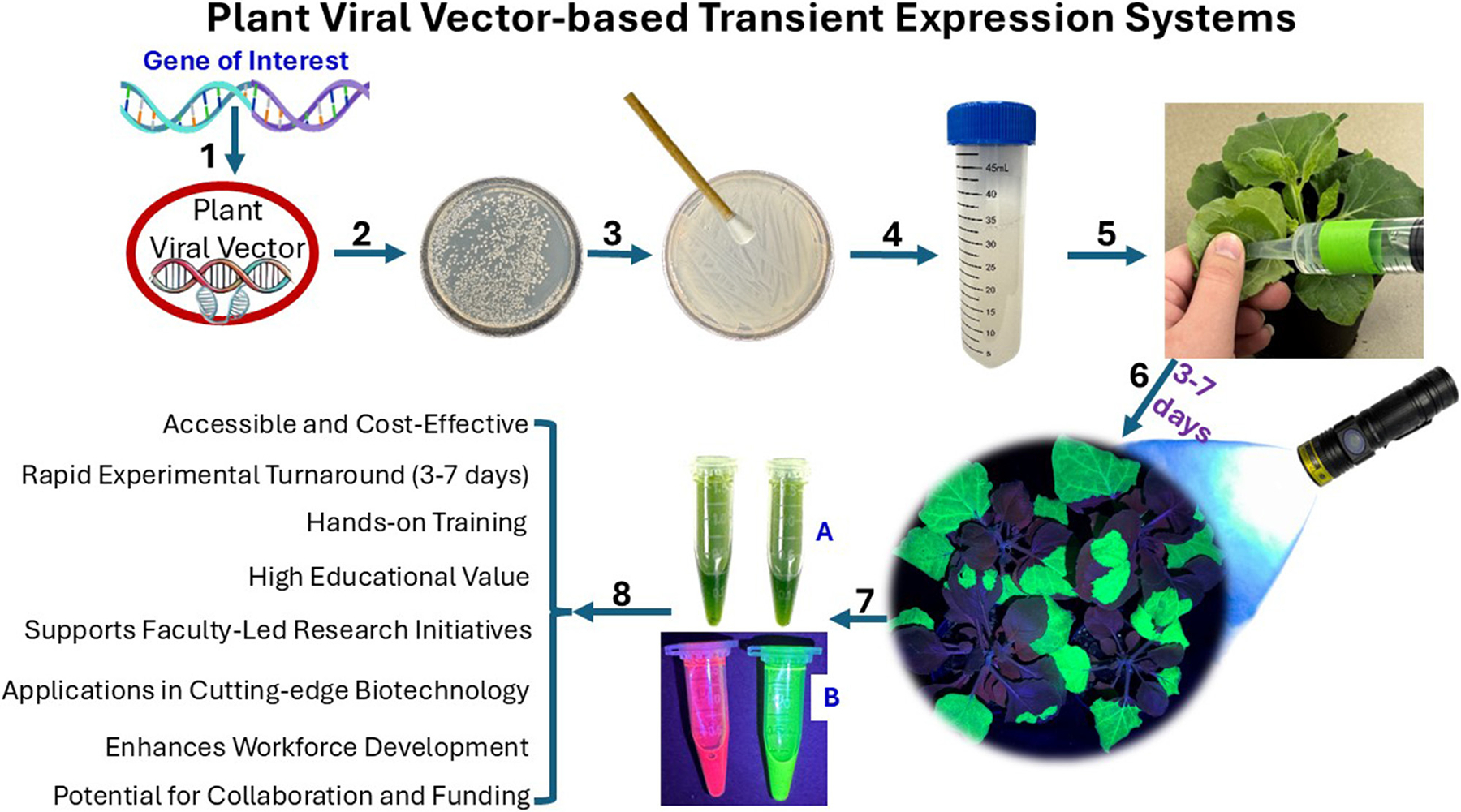
Workflow and applications of plant viral vector-based transient expression in plants. This figure illustrates the stepwise procedure and key advantages of using a plant viral vector system for transient gene expression. Step 1: A gene of interest is inserted into a plant viral vector. Steps 2–4: The recombinant vector is introduced into *Agrobacterium tumefaciens*, which is then cultured and prepared for plant infiltration. Step 5: The *Agrobacterium* suspension is delivered into plant leaves using syringe-based agroinfiltration. Step 6: Gene expression is typically observed within 3–7 days and detected via fluorescence imaging, often using reporter proteins such as GFP. Steps 7–8: The expressed proteins can then be extracted from the infiltrated tissue and analyzed for a variety of downstream applications, depending on the research or instructional objective—such as protein characterization, functional assays, or synthetic biology prototyping.

Plant viral vector-based transient expression systems offer a compelling alternative. Using engineered plant viruses—such as Tobacco Mosaic Virus (TMV, Santoni et al., 2024), Potato Virus X (PVX, Ariga et al., 2020), or geminivirus-based vectors like Bean Yellow Dwarf Virus (BeYDV, Diamos et al., 2020)—these platforms can deliver genes of interest in plant tissues without genomic integration or tedious sterile tissue culture process. This enables rapid, high-yield production of recombinant proteins within 3 to 7 days, depending on the vector used (e.g., as early as 3 days with BeYDV) (Vergish et al., 2024) (Figure 1).

Transient expression systems are now recognized as reproducible, scalable platforms for applications including functional genomics, enzyme engineering, and plant-based biopharmaceutical development (Golubova et al., 2024). By integrating transient expression systems into undergraduate research and coursework, PUIs can significantly broaden student engagement in modern plant biotechnology.

A comparison of transient and stable transformation methods is provided in the Table 1.

## 2 Advantages of plant transient expression for PUIs

Plant transient expression systems can generate observable research outcomes within a week, making them particularly well suited for implementation at PUIs, where semester-based timelines often limit the scope of experimental work (Figure 1).

### 2.1 Low-cost and accessibility

In recent decades, plant transient expression technologies have undergone substantial advancements in both methodology and accessibility. Early approaches—such as protoplast-based transient expression—were labor-intensive and yielded limited protein expression. These methods required the enzymatic removal of the plant cell wall to allow DNA uptake, resulting in low recombinant protein yields typically in the microgram to milligram range per kilogram of leaf fresh weight (LFW) (Kharel et al., 2024). Detection of expressed proteins, such as green fluorescent protein (GFP), often depended on the use of specialized, high-cost fluorescence microscopy, limiting their practicality for small-scale or resource-constrained institutions (Eidenberger et al., 2023).

Modern transient expression systems have addressed earlier limitations by adopting plant virus-derived vectors from viruses such as TMV (Santoni et al., 2024), PVX (Ariga et al., 2020), and BeYDV (Diamos et al., 2020). These improved systems allow for significantly higher expression levels, reaching milligram to gram quantities per kilogram of LFW. Genes of interest are typically delivered using *Agrobacterium tumefaciens* via simple syringe (without needle) or vacuum infiltration, enabling direct gene delivery into plant tissues without the need for tissue culture or sterile growth environments. For example, GFP expression can now be visualized in 3–7 days using an inexpensive handheld UV lamp (approximately $20 on Amazon), eliminating the need for costly fluorescence microscopes.

With only basic molecular biology equipment, PUIs can successfully implement these systems, supporting undergraduate education and faculty-led research in plant biotechnology.

### 2.2 Educational impact and rapid turnaround suitable for teaching and research

The rapid turnaround time of plant transient expression systems allows students to complete entire experimental workflows—such as gene cloning, agroinfiltration, protein expression, and characterization—within a single semester. This timeline supports iterative learning and provides immediate feedback, reinforcing students’ understanding of core molecular biology concepts and helping sustain their motivation.

These systems also allow students to explore advanced topics, including gene function, metabolic engineering, and plant-based biopharmaceutical production (Yao et al., 2015). Their incorporation into laboratory courses, capstone projects, and Course-based Undergraduate Research Experiences (CUREs) promotes active learning, critical thinking, and collaborative problem-solving (Buchanan and Fisher, 2022).

### 2.3 Applications in cutting-edge plant biotechnology

Plant transient expression systems are highly versatile and have proven utility across a range of modern applications. These platforms have been employed in the development of plant-based vaccines against infectious diseases such as COVID-19 (Jung et al., 2022), and in the production of therapeutic proteins like recombinant anti-vascular endothelial growth factor (anti-VEGF) antibodies, which are used in angiogenesis inhibition and cancer therapy (Soleimanizadeh et al., 2022).Their short expression timeline also makes them suitable for gene editing applications, where fast prototyping and validation are essential (Zhang et al., 2020; Yao et al., 2020). Negishi et al. (2024) demonstrated that geminivirus-derived replicons (GVRs) can enable transient expression of CRISPR/Cas9 components in apple (*Malus* × *domestica*) without transgene integration. This viral vector-based system achieved targeted genome editing in “Fuji” apple cells, offering a promising approach for transgene-free modification in highly heterozygous fruit crops.

Plants are natural producers of valuable bioactive compounds, yet many of these occur at low concentrations or are restricted to specific species, making large-scale production challenging. Beyond protein production and gene editing, transient expression systems also support metabolic engineering and biofortification efforts. These include the design of plants capable of producing enhanced levels of nutrients, vitamins, antioxidants, or novel secondary metabolites (Rodriguez-Concepcion and Daròs, 2022). Golubova et al. (2024) highlight the use of *Nicotiana benthamiana* as a synthetic biology platform, emphasizing its suitability for transient expression to prototype genetic circuits and reconstruct metabolic pathways. Similarly, Perez-Colao et al. (2025) provide a protocol demonstrating how *A. tumefaciens*-mediated transient expression in N. benthamiana can be used to efficiently assemble multi-step metabolic pathways—such as carotenoid biosynthesis—in the cytosol. These advances enable rapid and high-yield biosynthesis of complex natural products without stable transformation.

The broad applicability of these systems also offers undergraduates opportunities to engage with modern topics in synthetic biology, plant-based pharmaceuticals, gene editing, and functional genomics, fostering scientific literacy, innovation, and meaningful contributions to the research community.

## 3 Implementation strategies for PUIs

To successfully implement plant virus vector-based transient expression at PUIs, only minimal but essential laboratory resources are required (Dahlberg et al., 2021; Waddell et al., 2021). These materials and equipment include:

- Basic molecular biology tools, such as micropipettes, centrifuges, PCR machines, and gel electrophoresis equipment.
- *Agrobacterium tumefaciens* cultures carrying plant expression vectors (e.g., GFP constructs). Free kits and standardized protocols are readily available from organizations like the International Society for Plant Molecular Farming (ISPMF), provided by the EU Horizon 2020 Pharma-Factory consortium (http://www.ispmf.org).
- Model plant systems such as *Nicotiana tabacum* or *N. benthamiana* are tobacco species that are easily grown and maintained under typical laboratory conditions.

Tobacco species serve as ideal model plants for transient expression studies at PUIs due to their non-food crop status, which reduces the risk of food chain contamination and mitigates public concern regarding genetically modified organisms (GMOs). Their compatibility with open laboratory environments and ease of cultivation further enhance their suitability for undergraduate research. Combined with the minimal infrastructure required for viral vector-based transient expression, these systems provide an accessible, safe, and effective platform for training students in molecular biology, synthetic biology, protein engineering, and plant-based biomanufacturing—often leading to publishable research outcomes (Dickey et al., 2017).

In semester-based laboratory courses, instructors can incorporate transient expression modules to teach key techniques such as gene optimization, DNA cloning, bacterial inoculation, protein purification, SDS-PAGE, and Western blotting. These experiences not only enhance learning outcomes but also provide students with marketable skills relevant to biotechnology and biomedical research.

**FIGURE 2:**
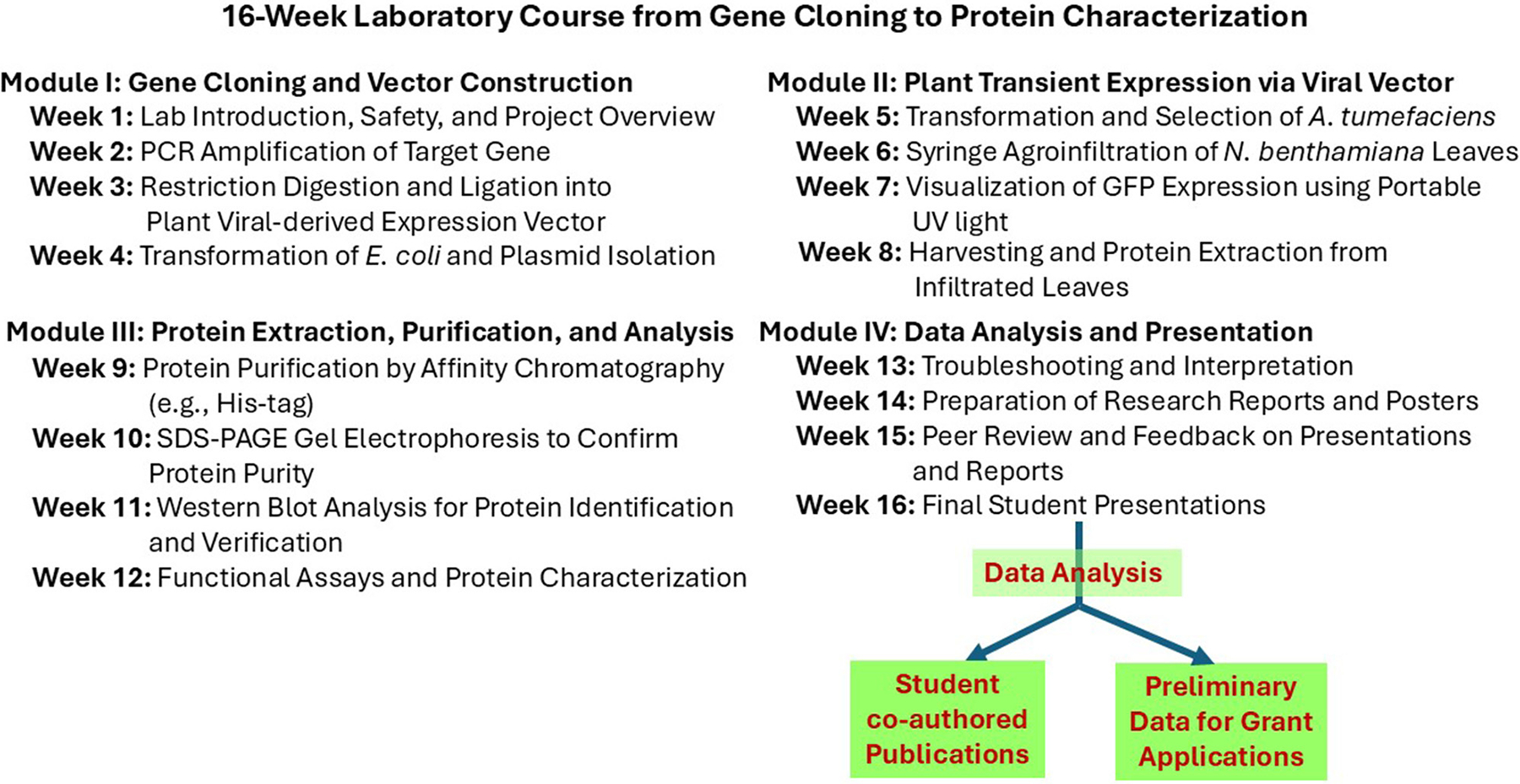
Overview of a 16-week laboratory course on gene cloning to protein characterization. This figure outlines a modular 16-week undergraduate laboratory course that guides students through gene cloning, plant-based protein expression using viral vectors, protein purification and analysis, and scientific communication. The course culminates in data analysis and student presentations, with potential outcomes including potential co-authored publications and preliminary data for research grants.

A well-designed 16-week lab course (Figure 2) incorporating transient expression can lead to tangible student achievements, including data suitable for presentations, manuscript preparation, or preliminary results for grant proposals—greatly enriching their academic and professional development.

Establishing and sustaining a plant transient expression platform at PUIs requires targeted external funding. Multiple federal, state, and private agencies offer grant opportunities focused on undergraduate research, especially in biotechnology, biomedical education, and hands-on student training.

At the federal level in the United States, the National Science Foundation (NSF) supports PUIs through programs such as Research in Undergraduate Institutions (NSF-RUI) and Research Experiences for Undergraduates (NSF-REU). The National Institutes of Health (NIH) also offers support through the IDeA Networks of Biomedical Research Excellence (INBRE) and Academic Research Enhancement Awards (AREA R15/R16). Additionally, the U.S. Department of Agriculture (USDA) provides funding for undergraduate research projects that emphasize skill development, tangible research outcomes, and student-authored publications.

Regional programs like the Kentucky INBRE (KY-INBRE) offer substantial support to PUIs advancing biotechnology education. At the University of Pikeville (UPIKE), KY-INBRE funding—including Research Project Awards (RPA) and CUREs—has enabled successful implementation of plant transient expression platforms in biotechnology curricula. This support has provided students with valuable hands-on training in molecular biology and biotechnology techniques.

To advance biotechnology education and cultivate regional talent in Eastern Kentucky and the Appalachian area, the University of Pikeville (UPIKE), a Primarily Undergraduate Institution, launched a biotechnology laboratory course in spring 2024. Using a plant transient expression platform and basic laboratory infrastructure, the course was designed to provide students with authentic, research-based learning. Initially, students anticipated a traditional biology lab; however, just 3 days after agroinfiltration, they observed clear phenotypic changes—such as yellowing or necrosis—linked to foreign gene expression. This immediate, hands-on experience helped students quickly develop an intuitive understanding of molecular biotechnology and the gene-to-phenotype workflow.

Real-time expression outcomes—such as GFP fluorescence or visible leaf phenotypes, depending on the gene of interest—enhanced students’ conceptual understanding of molecular biology and deepened their interest in research. The transient nature of gene expression prompted students to reflect on the dynamic behavior of biological systems, particularly how plants return to their original state as expression diminishes. For example, while GFP fluorescence may persist for 3–6 weeks post-agroinfiltration, the signal gradually fades as viral activity declines and tissue begins to senesce. Students also noted progressive yellowing in treated leaves due to viral infection, reinforcing the importance of detecting gene expression within specific time windows. These observations sparked valuable discussions around optimal sampling points, gene stability, and host responses—key concepts essential to effectively understanding and applying transient expression systems in plant biotechnology.

Students achieved proficiency in core course objectives, including experimental design, data interpretation, and scientific communication. These learning gains were reflected in a range of student-led scholarly outputs: eight students presented at the UPIKE Research Symposium and received Certificates of Excellence in Research; three students presented at the 6th Annual McNair-Ledford Undergraduate Research Symposium; four at the Kentucky Academy of Science Annual Meeting; and three at the KY-INBRE Second Annual Conference. Notably, three students received NIH/KY-INBRE travel awards to present at the 9th National IDeA Symposium of Biomedical Research Excellence (NISBRE) in Washington, DC—where they were awarded First Place for the Most Innovative Science Poster.

In addition, two gene sequences co-authored with students were successfully deposited in GenBank (accession numbers PV013638.1 and PQ432224), and students contributed to manuscript preparation, including this publication. These accomplishments reflect not only meaningful knowledge acquisition but also the development of research autonomy, technical proficiency, and scientific communication skills. Undergraduate co-authorship on this manuscript highlights a broader commitment to empowering students to engage meaningfully in the plant biotechnology community—especially within the context of Primarily Undergraduate Institutions (PUIs).

Taken together, these outcomes provide strong evidence that plant transient expression systems are not only technically feasible at PUIs but are also highly effective in fostering student learning, research engagement, and professional development. Continued strategic funding and collaborative partnerships will further enhance the research infrastructure at PUIs, promoting innovation and broader participation in plant biotechnology and biomedical research (Altman-Singles et al., 2023).

## 4 Conclusion and future perspectives

Virus vector-based transient expression systems represent a transformative approach for advancing plant biotechnology research and education at PUIs. Their low infrastructure requirements and streamlined protocols make them particularly well-suited for PUIs, facilitating a wide range of applications including capstone projects, independent studies, and innovative laboratory courses.

Looking forward, continued refinement and broader adoption of this technology will open new avenues in synthetic biology, plant-made pharmaceuticals, vaccine development, and metabolic engineering. PUIs are uniquely positioned to contribute to these emerging fields while preparing students to address real-world biotechnology challenges. By fostering collaboration and securing strategic funding, PUIs can expand their research capabilities and drive innovation in plant biotechnology and related disciplines.

## Figures and Tables

**TABLE 1:** Comparison of viral-based transient vs. stable transformation.

Feature	Viral-based transient expression	Stable transformation
Tissue culture process	No	Yes
Time to expression	3–7 days	Months to years
Integration into genome	No	Yes
Equipment required	Basic molecular lab tools	Growth chambers Greenhouse, etc.
Yield [Per kilogram of leaf fresh weight (LFW)]	High (Milligram to Gram)	Low (microgram to milligram)
Cost	Low	High
Accessibility for PUIs	High	Low
Suitability for students	High (Single semester)	Low (Long-term commitment)
